# Single-Nucleotide Variations, Insertions/Deletions and Copy Number Variations in Myelodysplastic Syndrome during Disease Progression Revealed by a Single-Cell DNA Sequencing Platform

**DOI:** 10.3390/ijms23094647

**Published:** 2022-04-22

**Authors:** Paul Lee, Rita Yim, Sin-Hang Fung, Kai-Kei Miu, Zhangting Wang, Ka-Chun Wu, Lester Au, Garret Man-Kit Leung, Victor Ho-Fun Lee, Harinder Gill

**Affiliations:** 1 Department of Medicine, School of Clinical Medicine, LKS Faculty of Medicine, University of Hong Kong, Hong Kong, China; pl85@hku.hk (P.L.); ritayim@hku.hk (R.Y.); lesterau@hku.hk (L.A.); garretleung@gmail.com (G.M.-K.L.); 2 School of Biomedical Sciences, Faculty of Medicine, Chinese University of Hong Kong, Hong Kong, China; shfung@link.cuhk.edu.hk (S.-H.F.); kelvinmiu@cuhk.edu.hk (K.-K.M.); wangzhangting@cuhk.edu.hk (Z.W.); 3 Department of Clinical Oncology, School of Clinical Medicine, LKS Faculty of Medicine, University of Hong Kong, Hong Kong, China; eymonwu@hku.hk (K.-C.W.); vhflee@hku.hk (V.H.-F.L.)

**Keywords:** myelodysplastic syndrome, hypomethylating agents, single-cell sequencing, clonal evolution

## Abstract

**Simple Summary:**

Myelodysplastic syndrome (MDS) is a myeloid neoplasm associated with complex clonal architecture. The application of single-cell sequencing is capable of revealing the clonal dynamics of MDS during disease progression and treatment resistance. This has advantages over bulk-tumor sequencing which is limited by its resolution. In this study, we evaluated two patients with MDS for the clonal dynamics of pathogenic mutations at the single-cell level of disease progression and resistance to hypomethylating agents (HMAs). There were two key observations. First, changes in the clonal heterogeneity of the pathogenic *FLT3-ITD*, *IDH2*, *EZH2*, or *GATA2* mutations was associated with disease progression and resistance to HMA. Secondly, disease progression and resistance to HMA was accompanied by the acquisition of copy number variations of *DNMT3A*, *TET2*, and *GATA2*.

**Abstract:**

Myelodysplastic syndrome (MDS) is a clonal myeloid neoplasm characterized by ineffective hematopoiesis, cytopenia, dysplasia, and clonal instability, leading to leukemic transformation. Hypomethylating agents are the mainstay of treatment in higher-risk MDS. However, treatment resistance and disease transformation into acute myeloid leukemia (AML) is observed in the majority of patients and is indicative of a dismal outcome. The residual cell clones resistant to therapy or cell clones acquiring new genetic aberrations are two of the key events responsible for drug resistance. Bulk tumor sequencing often fails to detect these rare subclones that confer resistance to therapy. In this study, we employed a single-cell DNA (sc-DNA) sequencing approach to study the clonal heterogeneity and clonal evolution in two MDS patients refractory to HMA. In both patients, different single nucleotide variations (SNVs) or insertions and deletions (INDELs) were detected with bulk tumor sequencing. Rare cell clones with mutations that are undetectable by bulk tumor sequencing were detected by sc-DNA sequencing. In addition to SNVs and short INDELs, this study also revealed the presence of a clonal copy number loss of *DNMT3A*, *TET2*, and *GATA2* as standalone events or in association with the small SNVs or INDELs detected during HMA resistance and disease progression.

## 1. Introduction

Myelodysplastic syndrome (MDS) is a myeloid neoplasm associated with complex clonal architecture and is characterized by ineffective hematopoiesis, cytopenia, dysplasia, and clonal instability [1,2]. Common chromosomal aberrations include loss of chromosomes 5q and 7 and gains of chromosomes 8, 19, and 21. Mutations of *SF3B1*, *TET2*, *ASXL1*, *SRSF2*, and *DNMT3A* are reported in more than 10% of all MDS [3,4]. These genetic lesions contribute to the clinical presentations, risk of progression to acute myeloid leukemia (AML), treatment response, and survival [4,5,6,7]. Prognosis is conventionally determined by the International Prognostic Scoring System-Revised (IPSS-R) and more recently by personalized prognostic models incorporating somatic mutations [4,6,7,8]. Resistance to the hypomethylating agents (HMAs) azacitidine (AZA) and decitabine (DEC) in higher-risked MDS (HR-MDS) represents a major unmet need [3]. Resistance to HMA indicates a poor prognosis with a median overall survival (OS) of only 4.3 months in HR-MDS [9].

Somatic mutations and epigenetic alterations contribute to HMA resistance and clonal evolution in response to treatment remains a challenge in the management of MDS [10,11,12]. The use of next-generation sequencing (NGS) may help to predict the response to HMA and guide the clinical use of novel targeted therapy such as *FLT3*, BCL2, *IDH1*, and *IDH2* inhibitors [13,14]. While bulk tumor sequencing may help to detect somatic mutations that predict treatment outcomes, the clonal heterogeneity characteristic of MDS remains a challenge for the detection of somatic mutations in small subclones that may confer treatment resistance. In small studies, the single-cell RNA (sc-RNA) sequencing of CD34+ cell has identified sub-populations with distinct gene expression profiles and demonstrated myeloid-biased hematopoiesis in patients with MDS [15,16]. This single cell-based approach increases the sensitivity and facilitates sub-clonal analysis. Nevertheless, CNV detection at the RNA level is still challenging as the bioinformatic inference of CNV lacks accuracy.

To study clonal evolution and changes in clonal architecture during the resistance to HMA at the single-cell level, we performed DNA sequencing in two patients with MDS who acquired resistance to HMA. In addition to studying SNV, INDEL, and CNV serially at single-cell levels, we also evaluated the sensitivity of single-cell sequencing technology in detecting rare sub-population of cells harboring pathogenic mutations.

## 2. Materials and Methods

### 2.1. Patients

Bone marrow samples of two patients with MDS treated with HMA were collected serially during response and subsequent resistance. Mononuclear cells were isolated by density separation prior to sequencing. This study was approved by the Institutional Review Board (IRB) of the University of Hong Kong and Hong Kong West Cluster and written informed consent was obtained.

Patient 1 was a 53-year-old man diagnosed with MDS with excess blasts-2 (MDS-EB-2). This patient was treated with AZA (100 mg/m^2^/day on days 1–7 per cycle) every 28 days. After 14 cycles of AZA, the patient with progressed secondary AML was treated with induction with daunorubicin (90 mg/m^2^/d on days 1–3), cytarabine (100 mg/m^2^/day on days 1–7), and the FLT3 inhibitor midostaurin (50 mg twice per day on days 8–21). He subsequently received one cycle of a high dose of cytarabine (3 g/m^2^/dose every 12 h for 4 doses) and midostaurin (50 mg twice per day on days 8–21). He relapsed again and was refractory to treatment with venetoclax (400 mg/day on days 1–28)—DEC (20 mg/m^2^/day on days 1–5) and Gilteritinib (120 mg/day) monotherapy. He succumbed to secondary refractory AML. Patient 2 was a 51-year-old woman with MDS-EB-2, who refused treatment at initial presentation, received AZA (100 mg/m^2^/day on days 1–7) every 28 days for 7 months after her initial diagnosis. This patient subsequently refused induction chemotherapy or allogeneic hematopoietic stem cell transplantation (HSCT) and continued six further cycles of AZA, after which this patient progressed to secondary AML.

### 2.2. NGS of the Bulk Tumor Sample

DNA was extracted from bone marrow samples serially using a DNA blood mini extraction kit. NGS was performed serially using a 69-gene customized myeloid panel as previously described in the Supplementary Methods [17,18]. 

### 2.3. Targeted sc-DNA Sequencing

Sc-DNA sequencing was performed using the Tapestri Single-cell DNA AML Panel Kit (Mission Bio, San Francisco, CA, USA) according to the manufacturer’s protocol. Sequencing data were processed using Mission Bio’s Tapestri Pipeline V2.0. This pipeline comprised the following key steps: (1) adapter-trimming using Cutadapt, (2) reference genome alignment to hg19, (3) cellular barcode demultiplexing, (4) cell-based genotype calling using GATK/Haplotypecaller. Detection of *FLT3*-ITD was performed using additional scripts provided by Mission Bio and details were as described in the Supplementary Methods.

### 2.4. Data Sharing

All high-throughput sequencing data supporting the findings of this study were deposited in the Sequence Read Archive (SRA) with the BioProject number PRJNA748569. The sc-DNA raw reads were deposited to SRA under the accession codes SRR15209068, SRR15209069, SRR15209070, SRR15209071, SRR15209072, SRR15209073, SRR15209076, SR15209077, and SRR15209078. The bulk tumor sequencing raw data were deposited under the accession codes SRR15209074, SRR15209075, SRR15209079, and SRR15209080, respectively.

## 3. Results

### 3.1. Mutation Dynamics on Bulk Tumor Sequencing and Cytogenetics

Two patients with MDS were first studied by bulk sequencing of the diagnostic bone marrow and a serial reassessment of bone marrow samples. Patient 1 with MDS-EB-2 had a 12% blast at diagnosis, and the karyotype was normal. Bulk sequencing using a 69-gene custom panel detected 37 variants at diagnosis with the major pathogenic mutation being FLT3-ITD *with a VAF of 50% (Supplemental File S1). After 14 cycles of AZA (18 months)*, Patient 1 displayed a reduction in *the* mutation signature of *FLT3*-ITD VAF to 15.18% and a change in the list of variants was detected. Among the variants, the pathogenic *IDH2* R140H mutation was detected with a VAF of 30.64%. The patient subsequently progressed to secondary AML and was treated with induction with daunorubicin, cytarabine, *and* midostaurin. He achieved complete remission with incomplete hematologic recovery (CRi). He subsequently received one cycle of *high*-dose cytarabine and midostaurin. *He relapsed again on the 21 month* and was refractory to treatment with venetoclax—DEC and Gilteritinib monotherapy while the bulk tumor sequencing no longer detect any FLT3-ITD or IDH2 mutation. He succumbed secondary to refractory AML.

Patient 2 with MDS-EB-2 was presented with 13% bone marrow blasts at diagnosis and a complex karyotype as following: 46,XX, add (1) (p11), add (5) (q11.2), add (6) (p23), −8, + mar [11]/46, XX, add (1) (p11), add (5) (q11.2), add (6) (p23),−8,+ r [2]/46, XX [6]. Bulk sequencing showed two frameshift mutations of *KMT2A* (M2894fs or G2895fs) and *KMT2D* (G2346S) at a VAF of 17% and 45.92%, respectively (Supplementary File S1). These mutations were predicted to be functionally disruptive. This patient, who refused treatment at initial presentation, started treatment with AZA 7 months after diagnosis. Bulk tumor sequencing performed at this time point showed the negative KMT2A mutation while the *KMT2D* mutation persisted. The bone marrow examination at the start of treatment showed a bone marrow blast percentage of 17%. After three cycles of AZA, the bone marrow blast percentage was reduced to 13% but bulk sequencing was not performed due to limited sample availability. After six further cycles of AZA, this patient progressed to secondary AML with a bone marrow blast percentage of 21%. At progression to secondary AML, the karyotype showed the following: 46, XX, add (1) (p11), der (5) t (1;5) (p31;q11.2), ad1d (6) (p23), -8, +mar [13]/46,XX.

### 3.2. Clonal Architecture of SNVs and INDELs on sc-DNA Sequencing

**Figure 1:**
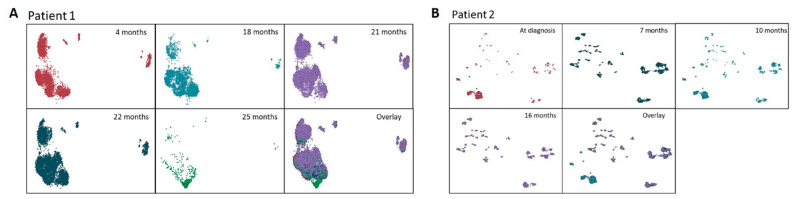
Clonal heterogeneity during treatment by density-based spatial clustering of cells with noise (DBSCAN) based on the allelic burden of SNV and INDELs allele burden in 2 patients with MDS. (**A**): Patient 1 at 4 months (after 4 cycles of AZA), at 18 months (at progression to secondary AML after 18 cycles of AZA), at 21 months (at relapse after the first cycle of midostaurin and high-dose cytarabine consolidation), at 22 months (non-remission to one cycle of venetoclax-DEC) and at 25 months (non-remission after 3 months of Gilteritinib); (**B**): Patient 2 at diagnosis, at 7 months (before the first cycle of AZA), at 10 months (after 3 cycles of AZA), and at 16 months (at progression to secondary AML after 9 cycles of AZA).

**Figure 2:**
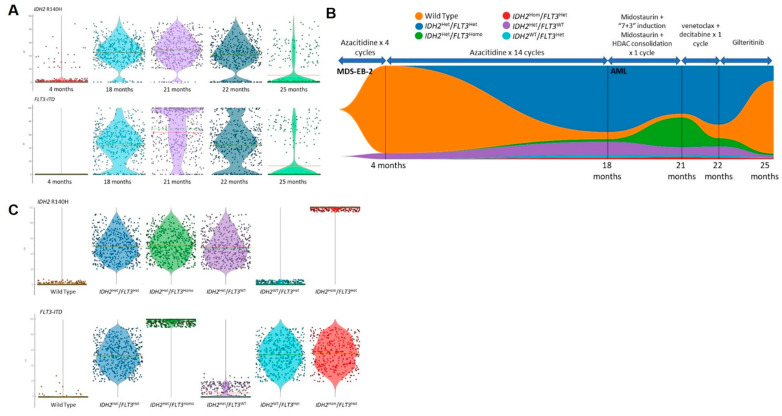
Clonal evolution of *IDH2* R140H and *FLT3*-ITD mutations in Patient 1. (**A**): Overall VAFs for IDH2 R140H and FLT3-ITD across different time points evaluated; (**B**): Fish plot showing the evolution of WT, FLT3-ITD, and IDH2 R140H clones serially with treatment; (**C**): VAFs of IDH2 R140H and FLT3-ITD mutations at the sub-clonal level. Red and green lines on the violin plots represent median and mean VAFs, respectively.

For Patient 1, a total number of 34,931 cells from five time points (8573, 3751, 7533, 14080, and 994 cells for each time point, respectively) were sequenced using sc-DNA sequencing platform. There were 98 variants detected across the five time points with good panel uniformity and an overall coverage of 31×–600× per cell per amplicon (Supplemental File S2). Due to poor cell viability, the diagnostic sample of Patient 1 did not complete sc-DNA sequencing and sc-DNA analysis started after four cycles of AZA. The clustering analysis of pooled variants showed multiple cell clusters with a gradual reduction in clonal heterogeneity and dominance by 1 to 2 clusters during the treatment course. Despite having fewer cells successfully sequenced at 25 months from diagnosis, the dominant cell clone showed unique features compared with previous time points (Figure 1A). Among the list of variants, two pathogenic mutations were identified with high genotype quality (GQ) (Supplemental Files S3 and S4), and a dynamic mutation burden was observed as follows: (1) chr15:90631934:C/T (IDH2:p.R140H) and (2) chr13:28608262: /CTGAAATCAACGTAGAAG (*FLT3*-ITD) (Figure 2A). The average VAF of IDH2 remained stable at approximately 40% and decreased significantly at 25 months with a minor fraction of cells retaining the mutation. On the other hand, the VAF of FLT3-ITD mutation fluctuated between 18–22 months, when Patient 1 received midostaurin with induction/consolidation chemotherapy, and venetoclax-DEC. The VAF of FLT3-ITD decreased significantly when Patient 1 received Gilteritinib. At the clonal level, 94% of cells were wild type (WT) during initial treatment with AZA, with a minor clone carrying a heterozygous IDH2 mutation (Figure 2B,C). During transformation into AML at 18 months from diagnosis (after 14 cycles of AZA), this clone expanded together with another clone carrying the double heterozygous for IDH2 and FLT3-ITD mutations. Other minor subclones with a homozygous mutation of either or both of the two genes were also detected, and the homozygous FLT3 mutant clones expanded despite treatment with midostaurin and induction/consolidation chemotherapy. When gilteritinib was started at 22 months, all mutant clones were suppressed and the WT clone became the major clone again. Nevertheless, none of the mutant clones were completely eradicated.

### 3.3. Rare Cell Clones with SNVs and INDELs Undetectable with Bulk Tumor Sequencing

**Figure 3:**
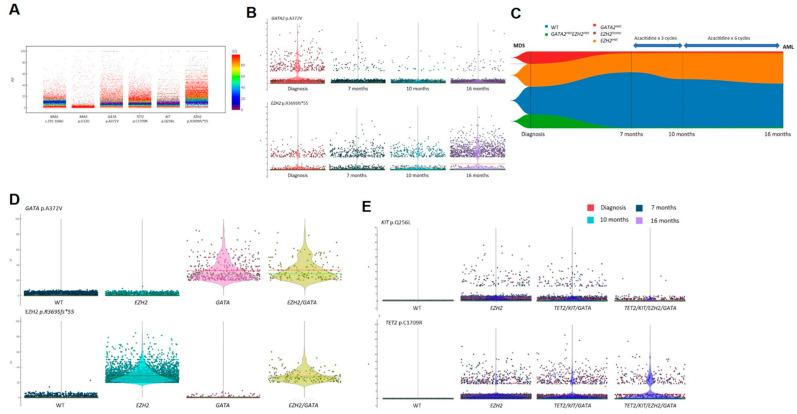
Detection and clonal evolutions of mutations in Patient 2. (**A**): VAFs and Quality of genotypes of rare pathogenic SNVs and INDELs which were present in <1% of total cells sequenced; (**B**): VAFs of major *GATA2* and *EZH2* mutations across different time points evaluated; (**C**): Fish plot evolution of WT, *GATA2*, and *EZH2* clones serially with treatment; (**D**): VAFs of *EZH2* and *GATA2* mutations at sub-clonal level; (**E**): VAFs of rare *KIT* and *TET2* mutations in association with *GATA2* and *EZH2* mutations across different time points. Red and green lines on violin plots represented median and mean VAF, respectively while the color of each cell represented corresponding time points.

For Patient 2, there were 130 variants detected in 37,710 cells sequenced across four time points (4692, 6092, 11,438, and 15,488 for each time point, respectively) with good panel uniformity and an overall coverage of 30×–76× per cell per amplicon (Supplemental File S5). Total variant clustering showed a distinct profile across the four time points (at diagnosis, 7, 10, and 16 months from diagnosis). Clustering homologies were observed between the diagnostic time point and at 10 months (after 3 cycles of AZA) where the bone marrow blast percentage was the same at 13% (Figure 1B). On the other hand, the clustering profile at 7 months (at the start of AZA) reassembled at 16 months post-diagnosis when she completed nine cycles of AZA. Among the variants evaluated for pathogenicity, the following four missense and two frameshift variants were shortlisted: (1) chr12-25398284-C-T (KRAS:p.G12D), (2) chr4-106196792-T-C (TET2:p.C1709R), (3) chr3-128200690-G-A (GATA2:p.A372V), (4) chr4-55569900-A-T (KIT:p.Q256L), (5) chr7-148515102-C- (EZH2:p.R369Sfs*55) and (6) chr12-25378716-GA g (KRAS: c.291-10del). The five variants of KRAS: p.G12D, TET2: p.C1709R, GATA2: p.A372V, EZH2: p.R369Sfs *55, and KRAS: c.291-10del were known pathogenic mutations, while KIT: p.Q256L was predicted to be tentatively pathogenic based on Varsome. A generally low VAF was observed for most variants but a low fraction of cells (<1% of total) showed high VAFs in KRAS, GATA, and KIT with high GQ (Figure 3A). For the GATA2, TET2, and EZH2 variants, a more diverse VAF heterogeneity was observed (Figure 3A). A longitudinal analysis of the GATA2 and EZH2 mutations showed that the VAF of the GATA2 mutation was highest at diagnosis and decreased after three cycles of AZA while the VAF of EZH2 mutation remained constant initially and increased after nine cycles of AZA (Figure 3B). A downstream clonal analysis of GATA2 and EZH2 showed that these mutations were derived from different heterozygous cell clones harboring either one or both of the mutations (Figure 3C). The two GATA2 mutant clones (GATA2^Het^ single mutant and GATA2^Het^/EZH2^Het^ double mutant clones) diminished at disease progression but remained as minor clones throughout the remaining time points while the smallest EZH2 homozygous clone was no longer detectable at disease progression (Figure 3C). When reviewing the rare mutations, there were minor fractions of GATA2 and EZH2 mutant cells that harbored the KIT or TET2 mutation. Most of these triple mutant cells persisted across all time points with no specific time points being enriched for these minor subclones (Figure 3E).

### 3.4. Clonal CNVs Associated with Pathogenic SNVs or INDELs during HMA Resistance

**Figure 4:**
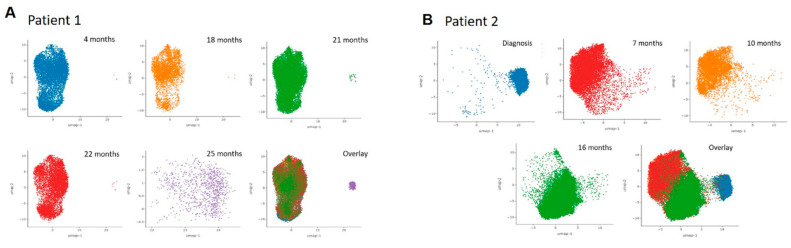
Heterogeneity of overall copy numbers during treatment by Principal Component Analysis (PCA) and Uniform Manifold Approximation Projection (UMAP) analysis. (**A**): Patient 1 at 4 months (after 4 cycles of AZA), at 18 months (at progression to secondary AML after 18 cycles of AZA), at 21 months (at relapse after the first cycle of midostaurin and high-dose cytarabine consolidation), at 22 months (non-remission to one cycle of venetoclax-DEC) and at 25 months (non-remission after 3 months of Gilteritinib); (**B**): Patient 2 at diagnosis, at 7 months (before the first cycle of AZA), at 10 months (after 3 cycles of AZA), and at 16 months (at progression to secondary AML leukemia after 9 cycles of AZA).

**Figure 5:**
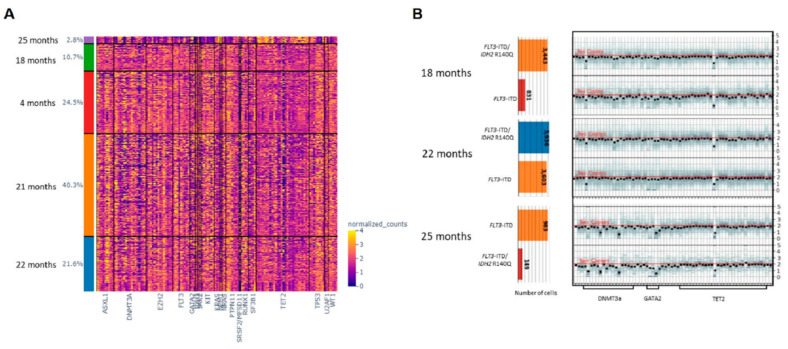
Co-occurrence of SNVs and short INDELs with CNVs in Patient 1. (**A**): merged CNV analysis of all time points; (**B**): Gradual copy number loss of DNMT3A, GATA2, and TET2 at 18 months (at progression to secondary AML after 18 cycles of AZA), at 22 months (non-remission to one cycle of venetoclax-DEC) and at 25 months (non-remission after 3 months of Gilteritinib).

**Figure 6:**
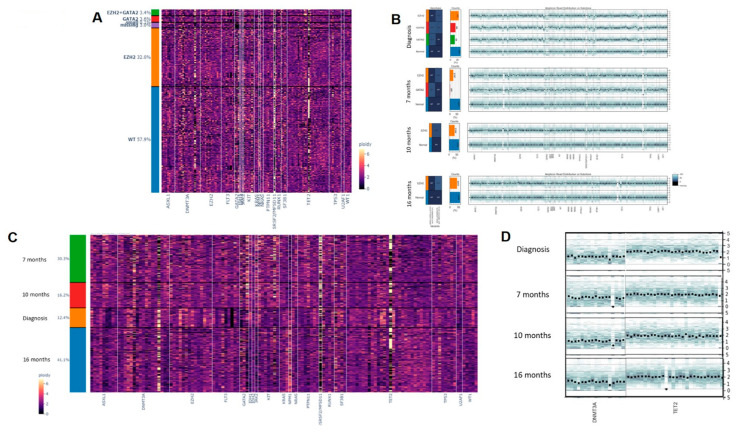
Co-occurrence of SNVs and short INDELs with CNVs in Patient 2 as independent cell clones. (**A**): merged CNV analysis of all time points; (**B**): CNV analysis at individual time points in association with *GATA2* p.A372V and *EZH2* p.R369Sfs*55 mutations; (**C**): Unbiased clustering of CNV analysis of all time points; (**D**): Gradual copy number loss of *DNMT3A* and *TET2* at 7 months (before the first cycle of AZA), at 10 months (after 3 cycles of AZA), and at 16 months (at progression to secondary AML after 9 cycles of AZA).

In addition to studying the dynamics of clonal SNV and INDEL, the CNVs of all 20 genes (127 amplicons) were studied and correlated with the IDH2 and FLT3-ITD mutations detected in Patient 1. In Patient 1, a cluster analysis of all amplicons pooled from all time points showed no significant differences during treatment with AZA and at progression to secondary AML (Figure 4A). An unbiased clustering analysis showed several CNVs in ASXL1, GATA2, DNMT3A, IDH1/2, KIT, and TET2. These CNVs were observed serially but ASXL1, DNMT3A, IDH1/2, and KIT deletion were enriched at 21 and 25 months when the patient failed treatment with midostaurin-based induction/consolidation, venetoclax-DEC and gilteritinib (Figure 5A). Despite observing a population of WT clone, loss of DNMT3A, GATA2, and TET2 were enriched in clones harboring FLT3-ITD and/or IDH2 mutations (Supplemental File S6). The magnitude of DNMT3A and GATA2 loss in FLT3 and/or IDH2 mutant clones also showed sequential changes as treatment resistance developed while TET2 CNV remained stable (Figure 5B). For Patient 2 where rare mutant clones were detected, a CNV analysis was performed to detect copy number abnormalities independent of the rare SNVs and INDELs to maximize sensitivity. Overall clustering in patient 2 showed distinct profiles at diagnosis compared with that at subsequent disease stages and homology was observed between 7 months (pre-treatment) and 10 months (after 3 cycles of AZA) from diagnosis. The profile at 16 months (at progression to secondary AML after 9 cycles of AZA) was somewhat intermediate between 7 and 10 months (Figure 4B). An analysis of all time points in association with GATA2 and EZH2 mutations did not lead to the detection of any large clonal specific CNVs compared with WT cells, except for the amplicon loss of TET2 exon 3 (106158314) in the GATA2^Het^ single mutant clone at 7 months diagnosis (Figure 6A,B). Consistent with the longitudinal analysis of GATA2 mutation, this clone was undetectable during treatment with AZA and at subsequent progression to secondary AML.

### 3.5. Clonal CNVs as Independent Events during HMA Resistance

To further detect clonal CNVs that were independent of GATA2 and EZH2 mutations in patient 2, unbiased clustering with pooled time points was performed. Putative deletions of TET2, ASXL1, DNMT3A, and potential artifacts of FLT3 and SRSF2 deletions were detected across multiple time points (Figure 6C). Both FLT3 and SRSF2 were later confirmed to be allele-dropouts due to poor PCR efficiency of amplicons and they were discarded from subsequent analysis (Figure 6C). To confirm the putative CNVs, repeated analyses normalized to cells with neutral polymorphisms were performed (Figure 6D). Among the three candidate CNVs, only DNMT3A and TET2 deletions were confirmed. Consistent drops of all DNMT3A amplicons were observed at 7 months (at the start of AZA) and at 16 months (at progression to secondary AML) together with a homozygous loss of TET2.

## 4. Discussion

In this study, we used a single-cell-based approach to detect mutations that were associated with disease progression in MDS and secondary AML. Using this high-resolution and sensitive method, the complex clonal heterogeneity of MDS cells during treatment resistance and progression was deciphered, demonstrating a distinct clonal architecture involving both SNVs, INDELs, and CNVs. In addition to the good correlation between the VAF on bulk tumor sequencing and the VAF of the sub-clones harboring *FLT3*-ITD and *IDH2* mutations, rare mutations that were below the detection limit of bulk tumor sequencing were detectable with sc-DNA sequencing. When gilteritinib was administered at 22 months to Patient 1, all mutant clones were suppressed; however, this may result in possible resistance to FLT3 inhibitors in several ways such as through the activation of survival pathways, cell adhesion to increase proliferation and adaptation of leukemic cells in bone marrow microenvironment by attenuating the expression of *CYP3A4* in bone marrow stromal cells resulting in activation to FLT3 inhibitor [19,20,21].

Although under-sequencing was observed at the last time point in Patient 1 and at diagnosis in Patient 2 compared with other time points, the high sensitivity to detect rare mutations of *KIT* was achieved when integrated analyses of all serial time points were performed. *KIT* and *TET2* mutations have been implicated in cellular differentiation and DNA methylation in MDS (1). We also demonstrated that this sc-DNA platform allows for the definitive determination of co-occurring mutations within the same cell.

Excluding the low fraction of allele-dropout events that led to false positive CNVs of *SRSF2* and *FLT3*, pathogenic losses of *DNMT3A* and *TET2* were detected in both patients. With *DNMT3A* showing a progressive loss in both patients, this suggested that continuous treatment with AZA or other DNMT inhibitors created a selection pressure for cell clones with *DNMT3A* loss or promoted the clonal acquisition of de novo *DNMT3A* mutations. While both *TET2* and *DNMT3A* were tumor suppressive in MDS, this study showed that *DNMT3A* loss is the primary or causative genomic abnormality to disease progression and treatment resistance [22,23]. In Patient 1, while *FLT3*-ITD clones were suppressed during potent FLT3 inhibition with Gilteritinib, disease remission was not achieved. In addition to the persistence of *IDH2* R140H clones and other rare cell clones, our study showed that copy number loss of *DNMT3A*, *TET2* and *GATA2* may play a role in resistance to FLT3 inhibitors.

## 5. Conclusions

In conclusion, this study demonstrated the clonal evolution of MDS at single-cell level treatment resistance to HMA. sc-DNA sequencing can uncover genetic mosaicism, and detect cells that are implicated in transformation into AML cell states which are crucial in underlining AML development and providing insights into treatment responses towards resistance development. SNVs and CNVs detected in this study clearly demonstrate their implication in cancer heterogeneity, and minor pathogenic clones that cannot be resolved by bulk tumor sequencing could be selected during prolonged treatments leading to drug resistance. The underlying evolutionary process involves not only SNVs and short INDELs but also acquired pathogenic CNVs of *GATA2*, *DNMT3A*, and *TET2* that are associated with HMA resistance. These CNVs could be coupled with SNVs or small INDELs of *FLT3* and *IDH2*. Nevertheless, the effect of these mutations on cellular differentiation and proliferation, and the expression of cell surface markers will require a multi-omic approach to simultaneously detect mutations and phenotypic changes at the protein level. Accurately identified clonal populations and reconstructing clonal phylogenies could be more effectively achieved with single cell analysis. The resolution of clonal heterogeneity in MDS may allow for better disease monitoring and the early detection of resistant clones. Resistant clones prior to therapy may also be detected. This will facilitate the design of combinatorial treatment approaches in patients with MDS. The low mutation burden of common mutations such as *FLT3*-ITD in AML could also be better resolved to determine if monotherapy would be a good option when sc-DNA sequencing is applied to detect the proportional of homozygous clones.

## Data Availability

All high-throughput sequencing data supporting the findings of this study were deposited in the Sequence Read Archive (SRA) with the BioProject number PRJNA748569. The sc-DNA raw reads were deposited to SRA under the accession codes SRR15209068, SRR15209069, SRR15209070, SRR15209071, SRR15209072, SRR15209073, SRR15209076, SR15209077, and SRR15209078. The bulk tumor sequencing raw data were deposited under the accession codes SRR15209074, SRR15209075, SRR15209079, and SRR15209080.

## Supplementary Materials

The following supporting information can be downloaded at: https://www.mdpi.com/article/10.3390/ijms23094647/s1. References [24,25,26,27,28,29,30,31,32,33,34,35,36] are cited in the Supplementary Materials.
