# Supplementary material for: Single-Nucleotide Variations, Insertions/Deletions and Copy Number Variations in Myelodysplastic Syndrome during Disease Progression Revealed by a Single-Cell DNA Sequencing Platform
Source: Int J Mol Sci. 2022 Apr 22;23(9):4647. doi: 10.3390/ijms23094647 (PMC9100947; doi:10.3390/ijms23094647)
Supplement: Supplementary file 1 [file ijms-23-04647-s001.zip › ijms-1674587-Supplemental Methods.pdf]

Supplemental Methods

Bulk tumor sequencing:

DNA was extracted from bone marrow samples serially using DNA blood mini extraction kit. NGS was performed serially using 69-gene customized myeloid panel as previously described[1, 2]. Enrichment of 69 genes was performed and they comprised *ABL1, ANKRD26, ACD, ATRX, BRAF, BCOR, BCORL1, ASXL1, CALR, CBL, CBLB, CDKN2A, CEBPA, CBLC, CREBBP, CSF3R, CUX1, DNMT3A, DDX41, ETV6, EZH2, FLT3, FBXW7, GATA1, GATA2, GNAS, GNB1, HRAS, IDH1, IDH2, JAK2, JAK3, IKZF1, KIT, KRAS, KMT2A, KDM6A, KMT2D, KMT2B, MPL, MYD88, NF1, NOTCH1, NPM1, NRAS, PDGFRA, PTEN, PTPN11, RAD21, PPM1D, RUNX1, ROBO1, ROBO2, SMC1A, SMC3, SETDB1, SF3B1, SETBP1, SETD2, SRSF2, SRP72, TERT, STAG2, TET2, TP53, U2AF1, WT1, ZRSR2* and *PHF6*. The enriched libraries were sequenced pair-ended on the Illumina MiSeq System (Illumina, San Diego, California, USA) followed by in-house analyses involving Trimmomatic and BWA [3, 4]. GATK and VarScan2 were used for variant calling and detection of *FLT3-ITD* was performed using PINDEL [5-7]. The resulting variants were annotated by ANNOVAR and SnpEff followed by manual evaluation [8, 9].

Targeted single-cell DNA (sc-DNA) sequencing:

Cryopreserved cells were thawed and counted before loading 35μL of cell at a concentration of 3500 cells/μL onto the Tapestri microfluidic cartridge. Cells were emulsified with lysis buffer and incubated at 50°C for 1 hour followed by thermal inactivation of the protease. The emulsion containing the lysates from protease-treated single cells was then microfluidically combined with targeted gene-specific primers, PCR reagents, and cell-identifying molecular barcodes beads using the same cartridge. Upon cell barcoding, the emulsion was amplified to incorporate the barcode identifiers into amplified DNA from the targeted genomic loci. The emulsions were then broken and the aqueous fraction was purified. Further downstream purification was performed using magnetic beads. Sample indexing PCR was performed and Illumina adaptor sequences underwent 10 additional cycles of PCR. The final libraries were purified and sequenced on Illumina NovaSeq with V4 150bp paired-end chemistry.

Analyzed data in h5 format were further analyzed using Mission Bio's Tapestri Insights using the local computer. SNV and short INDELs were filtered and analyzed using Mission Bio's Tapestri Insights software while CNV were analyzed using Mission Bio's Mosaic python package. In Tapestri Insights, high-quality cells and variants were filtered using the following criteria: (1) genotype quality score ≥ 30, (2) ≥10 reads per cell per amplicon, (3) variant allele frequency (VAF) for mutant genotype ≥ 15%, (4) variant genotyped in ≥ 50% of cells, and (6) ≥ 1% mutant cells detected. The variant significance was predicted using COSMIC, ClinVar, gnomad, and DANN prediction tools and the variant pathogenicity was predicted using

Varsome [10-15]. Variants clustering analysis was performed in a pooled manner using all time points. SNVs or INDELs with clinical implications confirmed from databases (ClinVar and dbSNP) and/or verified from previous bulk-tumor NGS were selected to assist identification of pathogenic cell clones.

For CNV detection, Clonal CNV analysis was performed following Mission Bio's mosaic Version 1.5 tertiary pipeline in python. Two analytic approaches were employed to complete this analysis with both involving defining a diploid clone as baseline according to selected SNV and INDEL genotype. The first approach defined the diploid clone using pathogenic genotypes determined from the previous step detecting CNV associated with pathogenic SNV or INDEL. Aiming to detect CNV independent from any pathogenic SNV or INDEL, the second analytic approach defined the diploid control clone by selecting SNV or INDEL predicted to be: (1) intronic, (2) functionally benign and/or (3) with stable VAF across most time points in ≥ 5% of cells carrying variants.

References:

1. Gill, H.; Leung, G. M. K.; Yim, R.; Lee, P.; Pang, H. H.; Ip, H. W.; Leung, R. Y. Y.; Li, J.; Panagiotou, G.; Ma, E. S. K.; Kwong, Y. L., Myeloproliferative neoplasms treated with hydroxyurea, pegylated interferon alpha-2A or ruxolitinib: clinicohematologic responses, quality-of-life changes and safety in the real-world setting. *Hematology* **2020,** 25, (1), 247-257.
2. Gill, H.; Ip, H. W.; Yim, R.; Tang, W. F.; Pang, H. H.; Lee, P.; Leung, G. M. K.; Li, J.; Tang, K.; So, J. C. C.; Leung, R. Y. Y.; Li, J.; Panagioutou, G.; Lam, C. C. K.; Kwong, Y. L., Next-generation sequencing with a 54-gene panel identified unique mutational profile and prognostic markers in Chinese patients with myelofibrosis. *Ann Hematol* **2019,** 98, (4), 869-879.
3. Li, H.; Durbin, R., Fast and accurate short read alignment with Burrows-Wheeler transform. *Bioinformatics* **2009,** 25, (14), 1754-60.
4. Bolger, A. M.; Lohse, M.; Usadel, B., Trimmomatic: a flexible trimmer for Illumina sequence data. *Bioinformatics* **2014,** 30, (15), 2114-20.
5. Van der Auwera, G. A.; Carneiro, M. O.; Hartl, C.; Poplin, R.; Del Angel, G.; Levy-Moonshine, A.; Jordan, T.; Shakir, K.; Roazen, D.; Thibault, J.; Banks, E.; Garimella, K. V.; Altshuler, D.; Gabriel, S.; DePristo, M. A., From FastQ data to high confidence variant calls: the Genome Analysis Toolkit best practices pipeline. *Curr Protoc Bioinformatics* **2013,** 43, 11 10 1-11 10 33.
6. Koboldt, D. C.; Zhang, Q.; Larson, D. E.; Shen, D.; McLellan, M. D.; Lin, L.; Miller, C. A.; Mardis, E. R.; Ding, L.; Wilson, R. K., VarScan 2: somatic

mutation and copy number alteration discovery in cancer by exome sequencing. *Genome research* **2012,** 22, (3), 568-76.
7. Ye, K.; Schulz, M. H.; Long, Q.; Apweiler, R.; Ning, Z., Pindel: a pattern growth approach to detect break points of large deletions and medium sized insertions from paired-end short reads. *Bioinformatics* **2009,** 25, (21), 2865-71.
8. Cingolani, P.; Platts, A.; Wang le, L.; Coon, M.; Nguyen, T.; Wang, L.; Land, S. J.; Lu, X.; Ruden, D. M., A program for annotating and predicting the effects of single nucleotide polymorphisms, SnpEff: SNPs in the genome of Drosophila melanogaster strain w1118; iso-2; iso-3. *Fly (Austin)* **2012,** 6, (2), 80-92.
9. Wang, K.; Li, M.; Hakonarson, H., ANNOVAR: functional annotation of genetic variants from high-throughput sequencing data. *Nucleic acids research* **2010,** 38, (16), e164.
10. Kopanos, C.; Tsiolkas, V.; Kouris, A.; Chapple, C. E.; Albarca Aguilera, M.; Meyer, R.; Massouras, A., VarSome: the human genomic variant search engine. *Bioinformatics* **2019,** 35, (11), 1978-1980.
11. Landrum, M. J.; Lee, J. M.; Benson, M.; Brown, G. R.; Chao, C.; Chitipiralla, S.; Gu, B.; Hart, J.; Hoffman, D.; Jang, W.; Karapetyan, K.; Katz, K.; Liu, C.; Maddipatla, Z.; Malheiro, A.; McDaniel, K.; Ovetsky, M.; Riley, G.; Zhou, G.; Holmes, J. B.; Kattman, B. L.; Maglott, D. R., ClinVar: improving access to variant interpretations and supporting evidence. *Nucleic acids research* **2018,** 46, (D1), D1062-D1067.
12. Sherry, S. T.; Ward, M.; Sirotkin, K., dbSNP-database for single nucleotide polymorphisms and other classes of minor genetic variation. *Genome research* **1999,** 9, (8), 677-9.
13. Smigielski, E. M.; Sirotkin, K.; Ward, M.; Sherry, S. T., dbSNP: a database of single nucleotide polymorphisms. *Nucleic acids research* **2000,** 28, (1), 352-5.
14. Karczewski, K. J.; Francioli, L. C.; Tiao, G.; Cummings, B. B.; Alfoldi, J.; Wang, Q.; Collins, R. L.; Laricchia, K. M.; Ganna, A.; Birnbaum, D. P.; Gauthier, L. D.; Brand, H.; Solomonson, M.; Watts, N. A.; Rhodes, D.; Singer-Berk, M.; England, E. M.; Seaby, E. G.; Kosmicki, J. A.; Walters, R. K.; Tashman, K.; Farjoun, Y.; Banks, E.; Poterba, T.; Wang, A.; Seed, C.; Whiffin, N.; Chong, J. X.; Samocha, K. E.; Pierce-Hoffman, E.; Zappala, Z.; O'Donnell-Luria, A. H.; Minikel, E. V.; Weisburd, B.; Lek, M.; Ware, J. S.; Vittal, C.; Armean, I. M.; Bergelson, L.; Cibulskis, K.; Connolly, K. M.; Covarrubias, M.; Donnelly, S.; Ferriera, S.; Gabriel, S.; Gentry, J.; Gupta, N.; Jeandet, T.; Kaplan, D.; Llanwarne, C.; Munshi, R.; Novod, S.; Petrillo, N.; Roazen, D.; Ruano-Rubio, V.; Saltzman, A.; Schleicher, M.; Soto, J.; Tibbetts, K.; Tolonen, C.; Wade, G.; Talkowski, M. E.; Genome Aggregation Database, C.; Neale, B. M.; Daly, M. J.; MacArthur, D. G., The mutational constraint spectrum quantified from variation in 141,456 humans. *Nature* **2020,** 581, (7809), 434-443.
15. Quang, D.; Chen, Y.; Xie, X., DANN: a deep learning approach for annotating the pathogenicity of genetic variants. *Bioinformatics* **2015,** 31, (5), 761-3.
