# Supplementary figures and images for: Single-Nucleotide Variations, Insertions/Deletions and Copy Number Variations in Myelodysplastic Syndrome during Disease Progression Revealed by a Single-Cell DNA Sequencing Platform
Source: Int J Mol Sci. 2022 Apr 22;23(9):4647. doi: 10.3390/ijms23094647 (PMC9100947; doi:10.3390/ijms23094647)

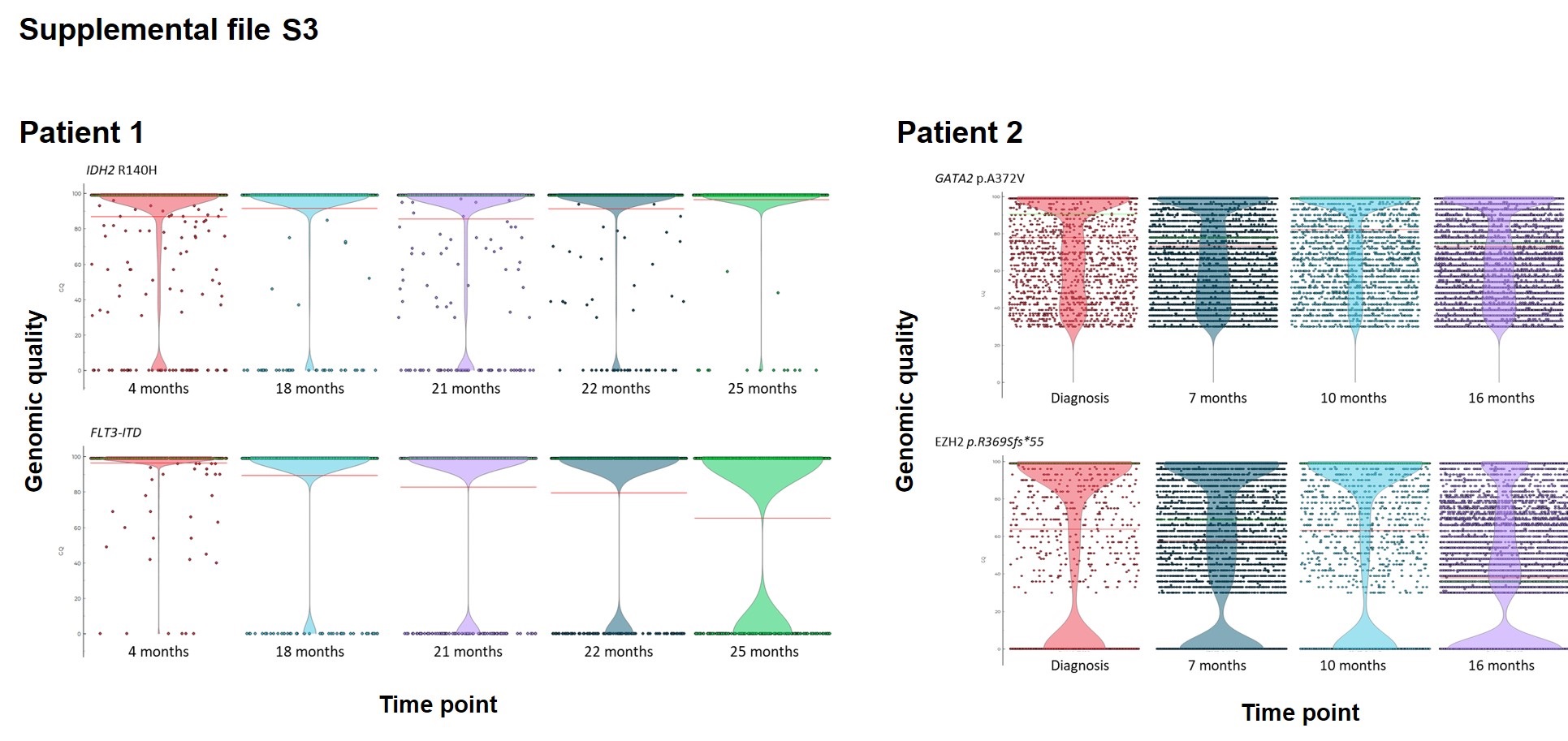

Supplement: Supplementary file 1 [file ijms-23-04647-s001.zip › Supplemental file S3.jpg]

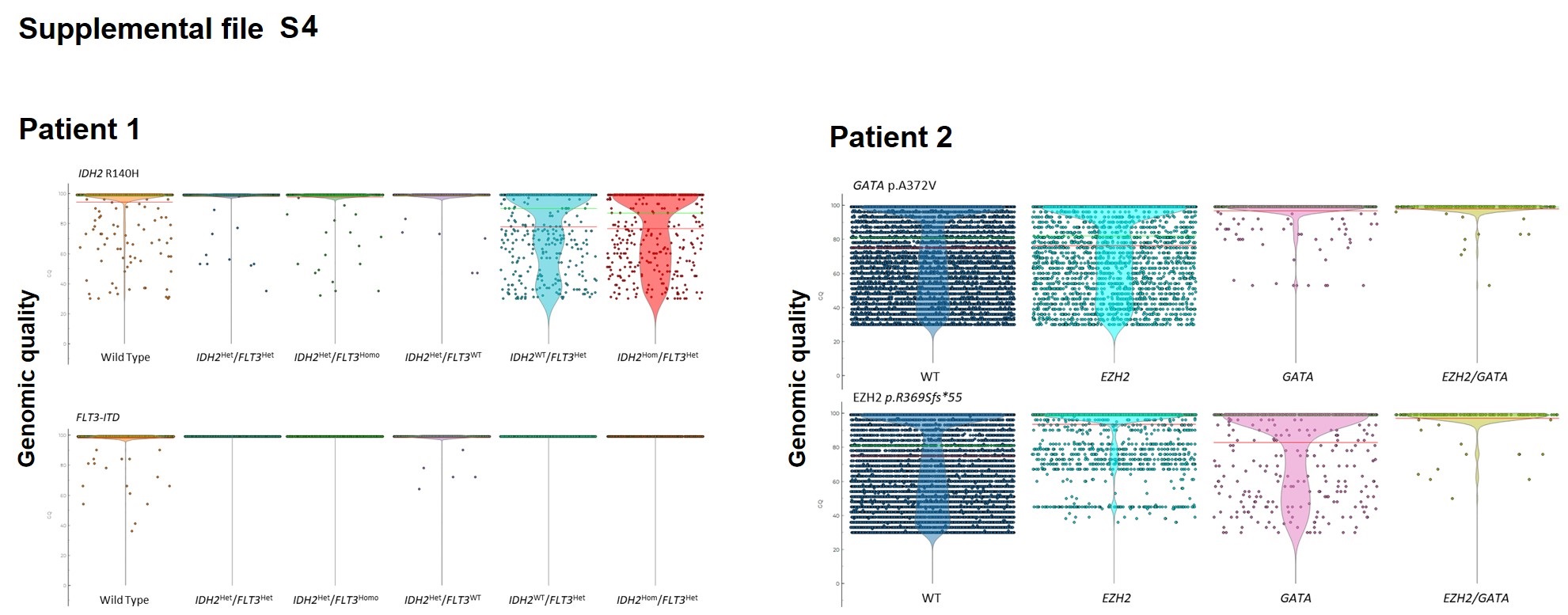

Supplement: Supplementary file 1 [file ijms-23-04647-s001.zip › Supplemental file S4.jpg]

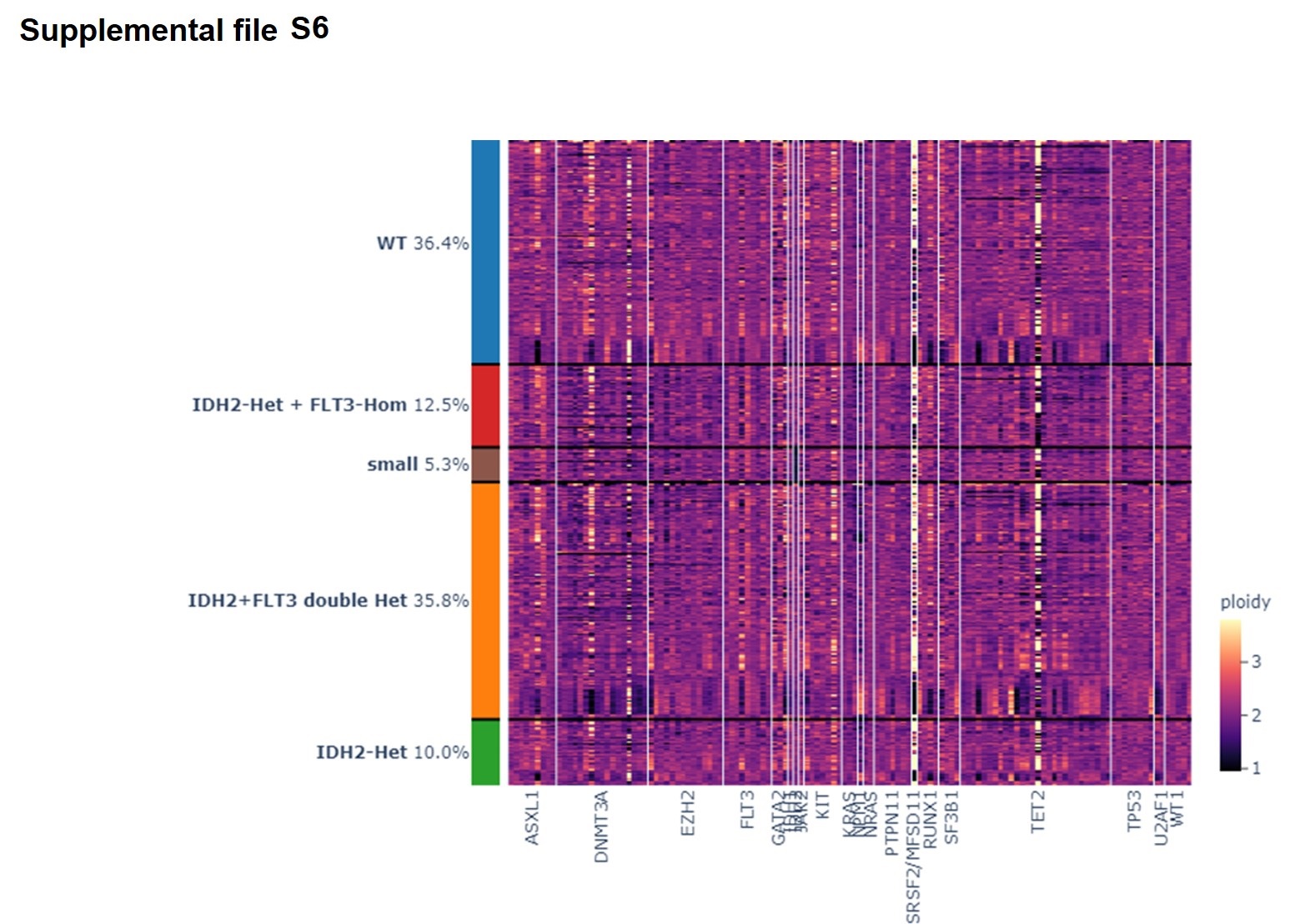

Supplement: Supplementary file 1 [file ijms-23-04647-s001.zip › Supplemental file S6.jpg]
